# Effect of physicochemical character differences on the genotoxic potency of kaolin

**DOI:** 10.1186/s41021-017-0075-y

**Published:** 2017-05-01

**Authors:** Tatsuya Kato, Tatsushi Toyooka, Yuko Ibuki, Shuichi Masuda, Masatoshi Watanabe, Yukari Totsuka

**Affiliations:** 1 Division of Carcinogenesis and Cancer Prevention, National Cancer Center Research Institute, 1-1 Tsukiji 5-chome, Chuo-ku, Tokyo, 104-0045 Japan; 2 Graduate School of Food and Nutritional Sciences, University of Shizuoka, 52-1, Yada, Shizuoka 422-8526 Japan; 3 Division of Materials Science and Engineering, Graduate School of Engineering, Yokohama National University, Hodogaya-ku, Yokohama, Japan; 4 Present Address: National Institute of Occupational Safety and Health, Nagao 6-21-1, Tama-Ku, Kawasaki, 214-8585 Japan

**Keywords:** Kaolin, Genotoxicity, Co-culture

## Abstract

**Background:**

Kaolin is white clay mineral with the chemical composition Al_2_Si_2_O_5_(OH)_4_, and many varieties of kaolins having different crystal structures are utilized in industrial, cosmetic and medical fields. To evaluate the effect of physicochemical character differences on the genotoxicity of kaolin, two types of kaolin, kaolin-S with smooth, sphere-shaped crystals, and kaolin-P with clusters of thin pseudohexagonal plates, were used in the study.

**Results:**

ICR mice were intratracheally instilled with the kaolins (0.05 and 0.2 mg/mouse), and comet assay was performed on their lungs. Both kaolins showed DNA damage in the lungs of the mice, however the DNA damaging potency was much higher with kaolin-P than that with kaolin-S.

In order to clarify the mechanisms for the different genotoxic potency, we examined the incorporation rate and ROS generation of these two types of kaolin in alveolar epithelial A549 and macrophage-like RAW264 cells, using flow cytometric (FCM) analysis. Kaolin-P showed a higher incorporation rate into the mammalian cells and ROS generation than that of kaolin-S. Especially, RAW264 cells aggressively incorporated kaolins, and generated ROS, whereas almost no ROS generation was observed in A549 cells. In addition, inflammatory cytokines were quantified, using the ELISA method, to understand further genotoxic potency differences of kaolins. Concentrations of interleukin-1β (IL-1β) and tumor necrosis factor-α (TNF-α) in the media were increased by exposure to both kaolins, but in the case of kaolin-P, these inflammatory cytokines were significantly elevated. Based on these findings, differences of genotoxic potency may contribute to incorporation rates into immune cells. Furthermore, it is likely that immune cells and epithelial cells might closely interact with each other for the appearance of genotoxocity in vivo. In order to clarify the interaction between epithelial and immune cells, A549 and RAW264 were co-cultured and RAW264 cells only were exposed to kaolins, then subsequently A549 was applied to FCM analysis and comet assay. DNA damage observed in the A549 cells markedly increased in the presence of kaolin-exposed RAW264 cells compared to the single culture.

**Conclusion:**

From these observations, it is suggested that mechanisms of kaolin genotoxicity against epithelial cells are through the activation of macrophage cells. Therefore, it is thought that interactions between epithelial and immune cells would be very important for evaluation of the genotoxicity of fine particulate matter. We also showed here that co-culture models of epithelial and immune cells could be used as suitable models for evaluation of lung genotoxicity of fine particulate matter, including nanomaterials, as in vivo mimicking systems.

## Background

Kaolin is a naturally occurring white clay mineral with the chemical composition Al_2_Si_2_O_5_(OH)_4_. A large amount of kaolin is primarily used in the paper industry both as a filler and as a coating for paper. Other applications of kaolin include use in the ceramics industry, cosmetics, and pharmaceuticals [1]. We have previously reported that kaolin showed genotoxic effects in in vitro and in vivo assay systems [2, 3]. Recently, there have been many reports that toxicity induced by fine particles is influenced by physicochemical differences such as size [4–9]. The chemical structure of kaolin is a two-layer silicate and is known to consist of a silica tetrahedral layer covalently bonded to an alumina octahedral layer through an apical oxygen atom [1]. Kaolinite is found as pseudo-hexagonal triclinic crystals and generally interacts between face-face, face-edge, and edge-edge surfaces to make an aggregate form [1]. Therefore, various sizes, zeta-potentials, and surface structures exist in the industrial mineral commodity of kaolins, and they are widely distributed for many intended uses. However the influences of physicochemical differences of kaolins on genotoxicity are not fully understood, yet. To verify the genotoxic effects of two kinds of kaolins representing different surface structures (one is smooth, sphere-shaped crystals named kaolin-S, and the other is clusters of thin pseudohexagonal plates named kaolin-P), here, we examined the DNA damaging potency with comet assay in in vivo and in vitro. The incorporation into mammalian cells and their ROS generation using flow cytometric (FCM) analysis were also investigated. In the present study, both kaolins showed DNA damage in the mice of lungs, however the DNA damaging potency was much higher for kaolin-P than that for kaolin-S. This potent genotoxicity of kaolin-P was also supported by the results obtained from analysis of the incorporation rate, ROS production and inflammatory cytokine generation. Furthermore, aiming to examine the mechanisms involved in the appearance of genotoxic potential differences in vivo induced by kaolin-S and -P, we conducted genotoxicity analysis using a co-culture system with lung epithelial A549 and macrophage-like RAW264 cells as an in vivo mimicking system. In the present study, possible mechanisms and importance of cell-cell interactions for genotoxicty induced by kaolin are also discussed.

## Methods

### Materials

Kaolin-S and kaolin-P were purchased from Takehara Chemical Industrial Co., Ltd. (Okayama, Japan) and Mineral and Pigment Solutions Inc. (NJ, USA), respectively. Kaolin-S is used for medical and pharmaceutical products and kaolin-P is used for industrial products. Both kaolins were suspended in saline (Otsuka Pharmaceutical Co. Ltd., Tokyo, Japan) containing 0.05% of Tween 80 (Nacalai Tesque, Kyoto, Japan) by sonication for 15–20 min, at a concentration of 2 mg/mL, as a stock solution. Crystal appearance observed under a scanning electron microscope (SEM) was done by A-KIT Corporation (Gifu, Japan). The size distributions of kaolins used in the present study was analyzed by dynamic light scattering (DLS) using FPAR-1000 (Otsuka Electronics Co., Ltd., Osaka) as described previously [2]. Physical characterization of particles such as zeta*-*potential was done by UBE Scientific Analysis Laboratory, Inc. (Yamaguchi, Japan). Type I agarose, low melting point agarose, dimethyl sulfoxide and Triton X-100 were bought from Sigma-Aldrich. Ethidium bromide was obtained from Merck (Darmstadt, Germany). Other chemicals were purchased from Wako Pure Chemical Industries (Osaka, Japan).

### Animals

Male ICR mice (8 weeks old) were purchased from Japan SLC (Shizuoka, Japan) and were acclimatized for 1 week. Food (CE-2 commercial diet: Japan Clea Co., Tokyo, Japan) and water were given freely. A conventional room was air conditioned at 23 °C with a light/dark (12 h/12 h) cycle. After quarantine for one week the experiments were conducted according to the “Guidelines for Animal Experiments in the National Cancer Center”.

### Evaluation of in vivo genotoxicity of kaolins

In order to evaluate the in vivo genotoxicity, alkaline comet assay on the lungs of mice was performed. Two doses of kaolins (0.05 and 0.2 mg per animal) were suspended in 0.1 mL of 0.05% Tween 80, then intratracheally instilled to mice (*n* = 5 for each dose) using a polyethylene tube under anesthesia of 4% halothane (Takeda Chemical, Osaka, Japan). The control mice (*n* = 5) were instilled intratracheally with 0.1 mL of the solvent alone, and non-treated mice were prepared to confirm the effect of Tween 80. Mice were sacrificed at 3 h after kaolin instillation and lungs were removed then subsequently minced and suspended with chilled homogenizing buffer and homogenized gently with a Dounce-type homogenizer on ice. Ten microliters of each cell suspension were mixed with 90 μl of 0.5% low melting point agarose and the mixture was spread on an MAS coated glass slide (Matsunami Glass Ind., Ltd, Osaka, Japan). The slide was immersed in lysing solution (2.8 M NaCl, 0.1 M EDTA-2Na, 0.01 M Tris-base, 0.2 M NaOH, 10% dimethyl sulfoxide, and 1% Triton X-100, and pH 10.0) and refrigerated at 4 °C overnight. Then, the slides were immersed in alkaline electrophoresis buffer (0.3 M NaOH, 1 mM EDTA-2NA) at 4 °C for 15 min to allow for DNA unwinding. Electrophoresis was performed for 15 min at 4 °C (25 V, 300 mA). The slides were neutralized with Tris buffer (0.4 M Tris-base, pH 7.5) at room temperature for 5 min, and dehydrated with ethanol to fix. The cells on the slide were stained with SYBR Gold® (10,000 × dilution; Molecular Probes, Eugene, OR) for 10 min, and washed with distilled water 3 times. After drying, comet images were analyzed using a fluorescence microscope (magnification 200×) equipped with CCD camera. Two slides (50 cells/slide) were prepared per mouse, and 100 cells were examined per mouse (Finally, 500 cells/10 slides were prepared from each treatment group). The percentage tail intensity was measured using Comet Assay IV (Perceptive Instruments, Ltd., Haverhill, UK). For evaluation of DNA damage, the slides were randomised and coded so that the treatment group was blinded to the scorer. The frequency of hedgehogs was not included in the data.

### Cell culture and treatment with kaolin

A549 (RIKEN Cell Bank, Tsukuba, Japan) and RAW264 (RIKEN Cell Bank) were maintained in MEM (Nacalai Tesque, Kyoto, Japan) supplemented with 10% fetal bovine serum, penicillin (100 U/mL) and streptomycin (0.1 mg/mL). The cells were cultured at 37 °C in 5% CO_2_. All experiments were performed with exponentially growing cells.

Kaolin stock solution was diluted with MEM at suitable concentrations, and sonicated for 10 min. A549 and RAW264 cells were treated with each kaolin at specific concentrations (20–200 μg/mL) and incubated for a predetermined time (30–180 min). After treatment, kaolin was removed and cells were harvested for each assay.

### Analysis of incorporation rates of kaolin

The incorporation rates of kaolin into mammalian cells were measured using the FCM analysis developed in previous reports [4, 10]. Briefly, A549 and RAW264 treated with several conditions of kaolin were trypsinized and suspended in fresh culture medium. Propidium iodide (PI; 0.5 μg/mL) was added to each cell suspension to determine the cell survival rates. The numbers of cells incorporating kaolin was analyzed with FACSCaliber (Becton Dickinson, Mountain View, CA). In the FCM analysis, forward-scattered (FS) light indicating cell size and side-scattered (SS) light indicating intracellular complexity were observed. The cells increasing SS were considered as material-incorporating cells, and the change in SS was an index of incorporation rates, as described previously [4, 10].

### Determination of intercellular ROS

Intercellular ROS in the mammalian cells were measured using FCM with 2′,7′-dichlorodihydrofluorescin diacetate (DCFH-DA) as described by Könczöl et al. with minor modifications [11]. DCFH-DA that has non-fluorescence can permeate the cell membrane, where subsequently it is hydrolyzed by unspecific esterases and converted into DCF that fluoresces in the presence of ROS. DCFH-DA was added into cell suspensions treated with kaolin at a final concentration of 20 μM, and incubated at 37 °C for 30 min. The cell suspensions were applied to FCM analysis, and the fluorescence was detected at an excitation wavelength of 485 nm and an emission wavelength of 530 nm. The numbers of cells increasing the fluorescence level were measured as the ROS generating cells.

### In vitro comet assay

DNA damaging potency of kaolin against mammalian cells was employed by comet assay using the same procedure described above (See in “Evaluation of in vivo genotoxicity of kaolins”) with some modification [2]. Briefly, ten μl of 8 × 10^3^ cells/mL cell suspension was mixed with 90 μl of 0.5% low melting point agarose and the mixture was spread on the MAS coated glass slide. After immersion in lysing solution, slides were incubated for 30 min at 37 °C with or without formadidopyrimidine-DNA glycosylase (FPG) protein (Sigma-Aldrich, St Louis, MO) in order to confirm oxidative DNA damage [12]. Thereafter, each slide was then placed in alkaline electrophoresis buffer to allow for DNA unwinding. After electrophoresis, the slides were neutralized and dehydrated, then the cells were stained with SYBR Gold®. DNA damage was measured by the same procedure for in vivo comet assay described above.

### Co-culture with lung epithelial and macrophage-like cells

**Scheme 1:**
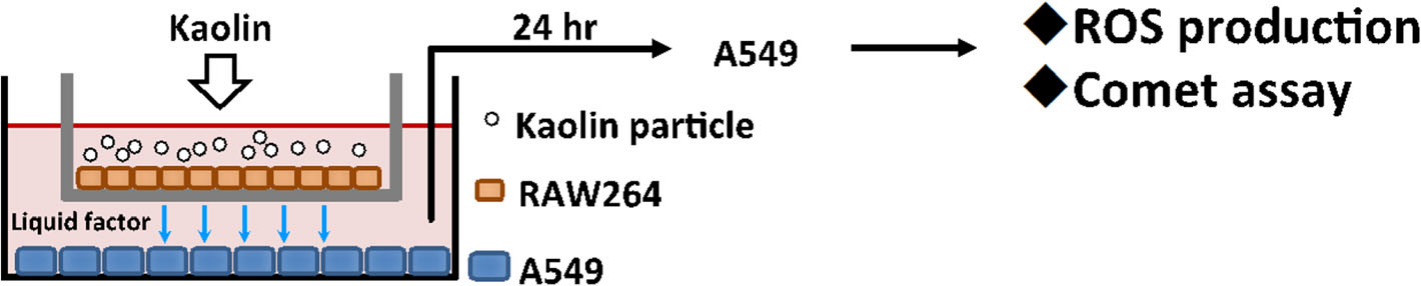
Co-culture model

To verify the interaction between epithelial and macrophage cells, A549 and RAW264 were co-cultured in the same culture medium (in vivo mimic system, Scheme 1). A549 cells were cultured in MEM with 10% FBS for 24 h, and subsequently RAW264 cells were seeded into cell culture inserts (pore size; 0.4 μm, high density, Greiner Bio-One Co., Ltd., St. Gallen, Switzerland). After culture for 24 h, only RAW264 cells were treated with 100 μg/mL of kaolin for 24 h, and A549 cells, parenchymal cells, were analysed by FCM (incorporation rates, intracellular ROS generation) and comet assay with FPG treatment.

### Measurement of inflammatory cytokines

Inflammatory cytokines in culture supernatant of RAW264 were measured. RAW264 cells were exposed to 100 μg/mL of kaolin for 24 h, subsequently IL-1β and TNF-α were quantified using an Immunoassay Kit, Mouse IL-1β (BioSource International, Camarillo, CA, USA) and Quantikine® Mouse TNF-α (R&D Systems, Minneapolis, MN, USA), respectively, according to manufacturers’ protocols. In brief, the supernatants obtained from culture media of RAW264 with or without kaolin treatment were transferred into 96 well plate and mixed with same volume of incubation buffer (for IL-1β) or assay diluent RD1-63 (for TNF-α), respectively. For detection of IL-1β, biotinylated anti-IL-1β solution was added and incubated for 90 min at 37 °C. The absorbance at 450 nm was measured after reaction with streptavidin-hoseradish peroxidase (HRP) and subsequent stabilized chromogen. In the case of TNF-α detection, monoclonal antibody for mouse TNF-α conjugated with HRP was subsequently reacted for 120 min and stabilized chromogen for 30 min in dark condition. Optical density at 450 nm (measuring) and 550 nm (correction) were measured and amounts of TNF-α were calucurated.

### Statistical analysis

The data from all studies except for comet assay are expressed as the mean ± standard deviations. The data were statistically compared using the Student’s t-test.

The data obtained from comet assay are expressed as mean ± standard errors. To test for significant differences of % tail intensity in the comet assay between a group treated with materials and an untreated group, Dunnett’s test after one-way ANOVA was used to evaluate the differences; *p* values lower than 0.05 were considered to indicate statistical significance.

## Results

### Characterization of kaolins

**Fig. 1:**
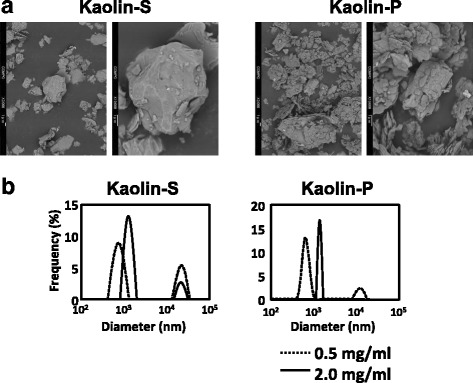
Crystal appearance and size distributions of kaolins. **a** SEM micrographs of kaolines obtained at E = 20 kV, X 3000 (*left*) and X 10,000 (*right*). **b** Size distributions of kaolins. Kaolins were suspended in saline containing 0.05% Tween 80 at a concentration of 0.5 and 2.0 mg/mL (*dashed line* and *solid line*, respectively) with 10 min sonication

To characterize and ascertain the properties of kaolins used in the present study, particle appearance, dispersed diameter and zeta-potential were determined. Figure 1a shows SEM images of two kinds of kaolin, kaolin-S and -P. The particles are smooth, sphere-shaped crystals for kaolin-S, and clusters of thin, pseudohexagonal plates for kaolin-P. The most abundant sizes of kaolin-S at doses of 0.5 and 2.0 mg/mL were 827.4 ± 186.2 and 1390.1 ± 226.3 nm, respectively (Fig. 1b). Those of kaolin-P were 700.0 ± 128.6 and 1488.3 ± 83.7 nm, respectively (Fig. 1b). The size distributions of these two kaolins were not different from each other. Moreover, the zeta-potentials were −8.29 mV for kaolin-S and −21.73 mV for kaolin-P.

### DNA damage in the lungs of mice induced by intratracheal instillation of kaolins

**Fig. 2:**
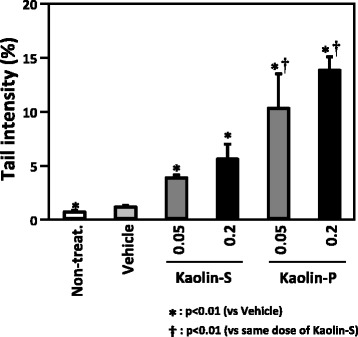
DNA damage in the lungs of mice intratracheally instilled with kaolins. ICR mice were intratracheally instilled with 0.05 and 0.2 mg/mouse of two kinds of kaolin. After 3 h of administration, the DNA damage in the lungs of the mice was analyzed using comet assay. The values represent the mean of five animals ± SD

In order to evaluate the effect of the physicochemical character of kaolins on DNA damaging potency, kaolins were intratracheally instilled to ICR mice, and comet assay was applied to the lungs. Both kaolins significantly induced DNA damage in the lungs of the mice, however the DNA damaging potency of kaolin-P was much stronger than that of kaolin-S (Fig. 2). At a dose of 0.2 mg/mouse, the tail intensities of kaolin-S and –P were 5.50 ± 1.38 and 13.74 ± 1.23, respectively. On the other hand, we examined the effects of different exposure times for not only 3 h but also 24 h. DNA damage induced by kaolin did not change either for 3 or 24 h (data not shown).

### Incorporation rates of kaolin in mammalian cells

**Fig. 3:**
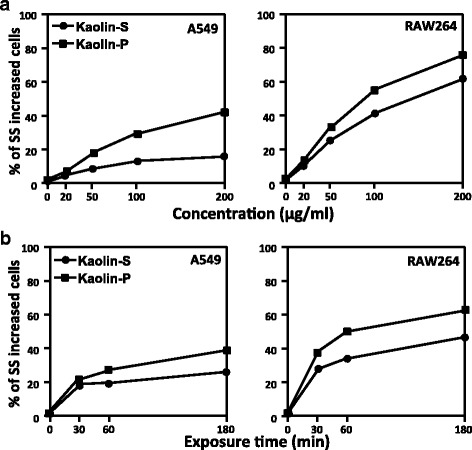
FCM analysis of incorporation rates of kaolins into mammalian cells. A549 and RAW264 cells were treated with two kinds of kaolin at several doses (20, 50, 100 and 200 μg/mL) for 30, 60 and 180 min. FCM analysis was applied to the cells to examine the incorporation rates. **a** Dose-dependent incorporation of kaolins (time duration was 180 min). **b** Time-dependent incorporation of kaolins (dose was 100 μg/mL)

Although the size distribution was almost the same, in vivo genotoxic potency was significantly different between kaolin-S and –P. In order to clarify the mechanisms of this finding, we examined incorporation rates of both kaolins using cultured mammalian cells. After exposure of both kaolins to A549 cells derived from lung epithelial cells, or RAW264 cells derived from macrophage-like cells, incorporation rates into the cells were analyzed by FCM analysis. Even though both kaolins were incorporated into both A549 and RAW264 cells in a dose- (Fig. 3a) and time- (Fig. 3b) dependent manner, the incorporation rate was greater in kaolin-P than in kaolin-S. Moreover, both kaolins were aggressively incorporated into RAW264 cells compared with A549 cells.

### Intercellular ROS generation

**Fig. 4:**
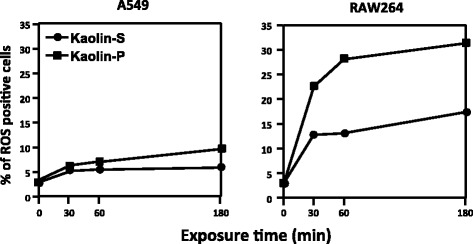
ROS generation in kaolin-exposed cells. A549 and RAW264 cells were exposed to two kinds of kaolin at a dose of 100 μg/mL for 30, 60 and 180 min, and subsequently, ROS generating cells were determined by using FCM with DCFH-DA

In order to verify the oxidative stress status, A549 and RAW264 cells were treated with both kaolins at a dose of 100 μg/mL for 30, 60 and 180 min. ROS generation was subsequently analyzed by using FCM with 2′,7′-dichlorodihydrofluorescin (DCFH) fluorescence. As shown in Fig. 4, the percentage of ROS generating cells increased up to 30 or 60 min then reached a plateau in RAW264 cells. Moreover, the rate of ROS positive cells was much greater with kaolin-P compared to kaolin-S, being in accordance with incorporation rates of these kaolins (Fig. 3b). In contrast, almost no ROS generating cells were observed in A549 cells exposed to either kaolin, whereas both kaolins were actually incorporated into A549 cells (Fig. 3b).

### Inflammatory cytokines released from RAW264

**Fig. 5:**
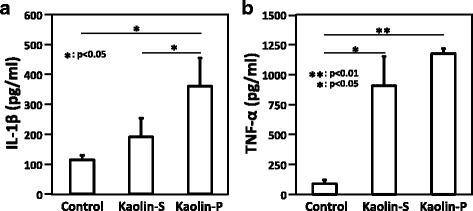
Generation of inflammatory cytokines. RAW264 cells were exposed to 100 μg/mL of kaolins for 24 h, and the concentrations of IL-1β and TNF-α in culture supernatants were measured by the ELISA method. Control represents treatment with saline containing 0.05% of Tween 80. **a** IL-1β. **b** TNF-α. The values represent the mean of 3 independent studies ± SD

When macrophage cells are exposed to exogenous materials, those cells were thought to release inflammatory cytokines such as interleukin-1β (IL-1β) and tumor necrosis factor-α (TNF-α) [13]. Thus, concentrations of IL-1β and TNF-α in culture supernatants of RAW264 were quantified by the ELISA method. When RAW264 cells were exposed to 100 μg/mL of kaolins for 24 h, the concentrations of IL-1β and TNF-α were significantly increased (Fig. 5). In the case of IL-1β, the value was significantly elevated by kaolin-P treatment rather than kaolin-S treatment. Similarly, the value of TNF-α concentration was elevated by kaolin-P rather than kaolin-S treatment, but not statistically significant.

### Interaction between epithelial cells and macrophage cells

**Fig. 6:**
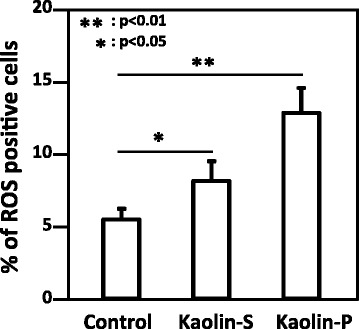
The effects on ROS generation in A549 cells caused by kaolin-incorporated RAW264. A549 and RAW264 cells were co-cultured and only RAW264 was exposed to kaolins. After 24 h, FCM analysis was applied to A549 cells with DCFH-DA. Control represents treatment with saline containing 0.05% of Tween 80. The values represent the mean of 3 independent studies ± SD

**Fig. 7:**
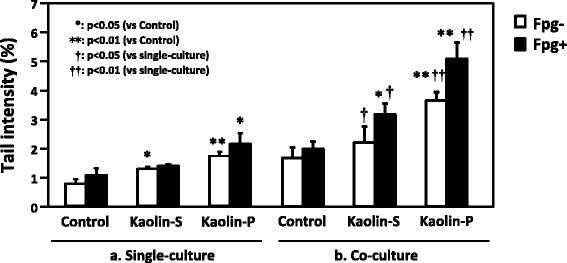
The effects on DNA damage in A549 cells co-cultured with kaolin-incorporated RAW264 cells. A549 was co-cultured with or without RAW264 cells, DNA damage in A549 cells was analyzed using comet assay with FPG protein. Kaolins were exposed to A549 cells (**a**) or RAW264 cells (**b**) at a dose of 100 μg/mL for 60 min. Control represents treatment with saline containing 0.05 of Tween 80. The open and closed columns represent the absence and the presence of FPG, respectively. The values represent the mean of 3 independent studies ± SD

In order to confirm the effect of kaolin-incorporating RAW264 cells on genotoxicity against A549, these two kinds of cells were co-cultured, as described in Materials and Methods (Scheme 1). Only RAW264 cells were exposed to 100 μg/mL of kaolins for 24 h, then ROS generation in A549 cells was analyzed by the FCM method. As shown in Fig. 6, ROS-generating A549 cells increased in the presence of kaolin-exposed RAW264 cells, and ROS generating potency was much greater with kaolin-P compared to kaolin-S. DNA damage in A549 was also examined by in vitro comet assay. In the case of single culture, the tail intensity observed in A549 cells was slightly elevated by exposure to both kaolins, whereas the tail intensity observed in A549 cells was significantly elevated in the co-culture system, and kaolin-P induced high DNA damage compared to kaolin-S in both culture systems (Fig. 7). Moreover, to determine the oxidative DNA damage, comet assay with formamidopyramidine-DNA glycosylase (FPG) protein was performed. DNA damage induced by both kaolins did not increase with FPG treatment under single-culture conditions (Fig. 7a). These results suggest that these kaolins were not capable of inducing ROS-dependent DNA damage in A549 cells, in a single-culture system. On the other hand, the tail intensity was largely increased in the presence of FPG treatment with both kaolins under co-culture conditions, therefore it is suggested that kaolin may induce oxidative DNA damage in epithelial cells through activation of macrophages.

## Discussion

We found that physicochemical characters, such as surface structure and zeta-potential, but not size, were different between two kinds of kaolin (kaolin-P and kaolin-S). It has been reported that toxicity induced by fine particles is influenced by their physicochemical differences [4–9], thus we examined and compared the genotoxicity of these two kaolins. In vivo DNA damaging potency was apparently different for each one, and kaolin-P especially showed much more potent genotoxicity. In order to understand the reason for the differences of genotoxic potency among these kaolins, we examined incorporation rate, ROS generation and inflammatory cytokine production by in vitro systems using A549 and RAW264 cells. We have previously reported that accumulation of nitrotyrosine was observed in macrophages and alveolar epithelial cells in the lungs of mice intratracheally instilled with kaolin, thus inflammation would be partly be involved in the appearance of genotoxicity [3]. Therefore, we used these two types of cultured mammalian cells, lung epithelial cells (A549) and macrophage-like cells (RAW264). In the FCM analysis, RAW264 cells strongly incorporated both kaolins compared to A549 cells. Among these different commercial products of kaolins, kaolin-P and –S, kaolin-P was more aggressively incorporated into both cells. Similarly, ROS generation was markedly observed in RAW264 cells whereas almost no ROS generation was observed in A549 cells. Especially for kaolin-P exposure to RAW264 cells, the percentage of ROS positive cells was significantly increased. Kaolin-P demonstrated markedly higher values in all assays in comparison with kaolin-S. There were no differences in the aggregation sizes of both kaolins, whereas the absolute value of zeta-potential, an indicator of surface electric charge, of kaolin-P was higher than that of kaolin-S. Thus, differences of surface electric charge may contribute to incorporation into cells. It is thought that nanomaterials that have high absolute zeta-potential values are likely to spread in suspension. Actually, the higher the negative charge of silver nanoparticles the stronger the toxicity in cultured cells [5]. These results predicted that kaolin-P is likely to spread in suspension relative to kaolin-S. Therefore, mammalian cells might more easily incorporate kaolin-P compared with kaolin-S. Actually, Suzuki et al. reported that the smallest titanium dioxide (TiO_2_) nanoparticle was most frequently incorporated into CHO cells [4]. However, detailed mechanisms for genotoxicity differences of these kaolins are not fully understand yet. Therefore further study is needed.

Many studies showed that genotoxic effects of fine particulate matter, including nanomaterials, were correlated with ROS generation [14–17]. It has been known that macrophages, immune phagocytes, produce ROS through the activation of NADPH oxidase (Nox) on endosomal membrane when macrophages incorporate exogenous materials [18–20]. Dostert et al. reported that ROS generation was observed in human macrophage THP-1 cells exposed to asbestos, and treatment with diphenylene iodonium and apocynin, Nox inhibitors, inhibited the ROS generation in THP-1 cells [21]. In the present study, we used RAW264 cells as immune system cells, therefore it is suggested that ROS generated by kaolin might be in the same manner as THP-1 cells. In contrast to RAW264 cells, lung epithelial cells, A549, showed little generation of ROS by either kaolin exposure (Fig. 4). Because limited incorporation rates of kaolin were observed in A549 cells, this would be the one of the reasons for the limited increase of ROS generation in A549 cells after exposure to kaolin. Moreover, we demonstrated inflammatory cytokines, such as IL-1β and TNF-α, were produced by kaolin-exposed RAW264 cells. It is reported that asbestos and silica nanoparticles activated Nlrp3 inflammasome in macrophage cells, subsequently the macrophage released IL-1β out of cells [21, 22]. Also, since *N*-acetyl-L-cysteine (NAC) and (2R, 4R)-4-aminopyrrolidine-2,4-dicarboxylate (APDC), a ROS inhibitor, decreased the release of IL-1β induced by asbestos, ROS is thought to be correlated with the generation of IL-1β through the activation of Nlrp3 inflammasome [21, 22]. On the other hand, Li et al. reported that intracellular ROS in A549 cell was increased by treatment with IL-1β [23]. Also, it was noted that IL-1β induced superoxide through the activation of Nox [24].

As mentioned above, ROS was considered to be an agent of nanomaterial-induced DNA damage. In fact, we previously demonstrated that kaolin induced oxidative DNA adducts such as 8-oxodG in the lungs of mice [3]. Therefore, we predicted that oxidative DNA damage in alveolar epithelial cells was induced by kaolin-phagocytized macrophage in an in vivo system. To verify the interaction between epithelial and macrophage cells, A549 and RAW264 cells were co-cultured and kaolin was exposed to only RAW264 cells. In the FCM analysis, intracellular ROS generation in A549 cells under co-culture conditions was significantly increased by both kaolins exposure, although almost no ROS generation was observed under single culture conditions. Furthermore, kaolin-P induced potent ROS generation in A549 cells compared to kaolin-S, thus these effects are thought to correlate with the amount of incorporation of these kaolins. In the case of comet assay under co-culture conditions, potent DNA damage was observed in A549 cells whereas DNA damage was slightly elevated under single culture conditions. Moreover, the DNA damage observed in the co-culture system with kaolin exposure was significantly increased by FPG treatment, but not in the single culture system. This observation indicated that ROS-dependent DNA damage was induced in A549 cells in the co-culture system with kaolin exposure. From these findings, we presumed that oxidative DNA damage in A549 was induced by kaolin-phagocytized RAW264 and may be mediated by inflammatory cytokines, including IL-1β and/or TNF-α. Actually, we revealed that kaolin-exposed RAW264 cells released IL-1β and TNF-α.

Supporting our findings, increased ROS production and inflammatory cytokine release were induced by nanomaterial treatment [11]. In addition, it has been reported that other immune phagocytic cells, neutrophils derived from bone marrow of C57BL/6 J mice, induced oxidative DNA damage in co-cultured A549 when the neutrophils were exposed to quartz particles [25]. Based on the above, as a genotoxic mechanism of fine particulate matter, including nanomaterials, it is likely that immune phagocytic cells intake fine particulate matter that then induces oxidative DNA damage in epithelial cells via release of inflammatory cytokines. Thus, considering the genotoxicity of fine particulate matter, interactions between epithelial and immune cells would be very important. However, most reports describing in vitro genotoxicity of nanomaterials used single culture systems. It is thought that single culture systems were not sufficient to evaluate the genotoxicity of nanomaterials. We showed in the present study, using a co-culture of epithelial and immune cells could be used as a suitable model for evaluating lung genotoxicity of nanomaterials as an in vivo mimicking system.

## Conclusions

We have demonstrated that two kinds of commercial products of kaolin with different physicochemical characters, such as surface structure and zeta-potential, revealed different genotoxic potency in vivo. Based on in vitro analysis, this genotoxic potency might be influenced by ease of uptake into immune cells. Mechanisms of kaolin genotoxicity against epithelial cells are suggested to be through the activation of macrophage cells. Namely, immune phagocytic cells ingested kaolin then induced oxidative DNA damage in epithelial cells via release of inflammatory cytokines, such as IL-1β and TNF-α. Therefore, it is thought that interactions between epithelial and immune cells would be very important for evaluation of the genotoxicity of fine particulate matter. We also showed here, co-culture models of epithelial and immune cells could be used as suitable models for evaluation of lung genotoxicity of fine particulate matter, including nanomaterials, as in vivo mimicking systems.
